# Authentication of a survival nomogram for non-invasive micropapillary breast cancer

**DOI:** 10.3389/fonc.2023.1156015

**Published:** 2023-07-12

**Authors:** Mingkun Zhang, Yuan Qin, Niuniu Hou, Fuqing Ji, Zhihao Zhang, Juliang Zhang

**Affiliations:** ^1^ Department of Thyroid, Breast and Vascular Surgery, Xijing Hospital, The Fourth Military Medical University, Xi’an, Shanxi, China; ^2^ Department of General Surgery, Eastern Theater Air Force Hospital of People’s Liberation Army (PLA), Nanjing, China; ^3^ Department of Thyroid Breast Surgery, Xi’an No.3 Hospital, The Affiliated Hospital of Northwest University, Xi’an, Shanxi, China

**Keywords:** invasive micropapillary breast carcinoma, overall survival, SEER, prognosis, nomogram

## Abstract

**Purpose:**

We aimed at establishing a nomogram to accurately predict the overall survival (OS) of non-metastatic invasive micropapillary breast carcinoma (IMPC).

**Methods:**

In the training cohort, data from 429 patients with non-metastatic IMPC were obtained through the Surveillance, Epidemiology, and End Results (SEER) database. Other 102 patients were enrolled at the Xijing Hospital as validation cohort. Independent risk factors affecting OS were ascertained using univariate and multivariate Cox regression. A nomogram was established to predict OS at 3, 5 and 8 years. The concordance index (C-index), the area under a receiver operating characteristic (ROC) curve and calibration curves were utilized to assess calibration, discrimination and predictive accuracy. Finally, the nomogram was utilized to stratify the risk. The OS between groups was compared through Kaplan-Meier survival curves.

**Results:**

The multivariate analyses revealed that race (*p* = 0.047), surgery (*p* = 0.003), positive lymph nodes (*p* = 0.027), T stage (*p* = 0.045) and estrogen receptors (*p* = 0.019) were independent prognostic risk factors. The C-index was 0.766 (95% CI, 0.682-0.850) in the training cohort and 0.694 (95% CI, 0.527-0.861) in the validation cohort. Furthermore, the predicted OS was consistent with actual observation. The AUCs for OS at 3, 5 and 8 years were 0.786 (95% CI: 0.656-0.916), 0.791 (95% CI: 0.669-0.912), and 0.774 (95% CI: 0.688-0.860) in the training cohort, respectively. The area under the curves (AUCs) for OS at 3, 5 and 8 years were 0.653 (95% CI: 0.498-0.808), 0.683 (95% CI: 0.546-0.820), and 0.716 (95% CI: 0.595-0.836) in the validation cohort, respectively. The Kaplan-Meier survival curves revealed a significant different OS between groups in both cohorts (*p*<0.001).

**Conclusion:**

Our novel prognostic nomogram for non-metastatic IMPC patients achieved a good level of accuracy in both cohorts and could be used to optimize the treatment based on the individual risk factors.

## 1 Introduction

In 2019, about 271,270 patients in the United States (US) were diagnosed with breast cancer, explaining about 30% of new diagnoses in women. Approximately 42,260 patients died from breast cancer (1). Invasive micropapillary carcinoma (IMPC) is a ductal lesion found in up to 8% of breast cancers (2–9). This histological subtype was first described in a randomized controlled trial by Fisher et al. in 1980, whereby 35 patients out of 1603 women were diagnosed with this subtype (10). Subsequently, Siriaunkgul and Tavassoli proposed a new official classification for IMPC in 1993 (11). Until 2003, IMPC was listed as a distinct pathological type in the classification of the World Health Organization, owing to its distinctive clinicopathology characteristics (12). IMPC is likely spreading via lymphatic vessels and lymph node metastasis than other subtypes. Guan et al. found that IMPC is more aggressive and has inferior disease-free survival and overall survival (OS) respect to invasive ductal carcinoma and ductal carcinoma in situ (13). However, some recent studies have shown that IMPC exhibits similar long-term survival outcomes compared to invasive ductal carcinoma (14, 15). As a result, there is a need to evaluate factors affecting survival in IMPC.

Nomograms are mathematical models used in medicine to describe how clinical variables are related to each other. The advantages of these models are that they are easy to use, intuitive, accurate, and reliable (16). As a result, nomograms are increasingly being used to predict survival in cancer and facilitate clinical decision-making (17, 18). However, there are currently no nomograms predicting the prognosis of non-metastatic IMPC. Therefore this study aimed at developing a prognostic nomogram for non-metastatic IMPC. The final nomogram was further verified on an external cohort of patients obtained from a Chinese hospital.

## 2 Materials and methods

### 2.1 Patients´ cohort

The data of patients diagnosed with non-metastatic IMPC were retrieved from the Surveillance, Epidemiology, and End Results (SEER) database. The program SEER * Stat (version 8.3.5) identified relevant patients from 2000 to 2014. We included female patients with a diagnosis of primary breast cancer according to the third edition of the International Classification of Diseases (ICD-O-3), with no distant metastasis (M0) and/or with a confirmed histological diagnosis of IMPC according to the histological/behavior code (ICD-O-3 Hist/behav, malignant) as training cohort. Patients aged below 20 or above 70 years, with bilateral breast cancer or unclear unilateral breast cancer, and/or those with incomplete follow-up (survival months = 0) were excluded. In addition, we excluded patients with missing clinical data, including information about race, grade, treatment (surgery, radiation therapy, and chemotherapy), TNM stage, estrogen receptor (ER) and progesterone receptor (PR) status.

According to previous inclusion and exclusion criteria, patients diagnosed with non-metastatic IMPC at the Xijing Hospital (Xian, China) between March 2006 and December 2016 were enrolled as part of the external validation cohort.

### 2.2 Ethics

The local Ethics Committee approved the study. We collected a written informed consent for all patients enrolled at the hospital.

### 2.3 Covariates and endpoints

The demographic data (ethnicity, age at the diagnosis and marital status), clinical data (grading, staging, ER and PR status and regional nodes), treatments (surgery, chemotherapy and radiotherapy) and follow-up information were retrieved from the SEER database. Unmarried people were defined as divorced, separated, widowed or single (having a domestic partner or never married). OS was the main primary endpoint, designated as the survival in months to all-cause mortality.

### 2.4 Construction of the nomogram

The categorical variables are expressed as proportions and frequencies. The Chi-square and Fisher’s exact tests compared the baseline categorical variables between training and external validation cohorts. The continuous variables are described as median and interquartile range (IQR). The Student’s t and non-parametric Mann-Whitney U tests made comparisons between quantitative variables of training and external validation cohorts. The Backward method was used for univariable and multivariable Cox analysis. Variables with P < 0.05 in univariable Cox analysis were incorporated into multivariable Cox analysis to determine independent prognostic factors of the control cohort. A nomogram was established to predict OS at 3, 5 and 8 years by integrating all independent prognostic factors. The Kaplan-Meier method was performed to calculate the survival rate, and the Log-rank test was applied to compare the differences between the curves.

### 2.5 Nomogram´s discrimination and calibration

The nomogram’s ability to discriminate between different survival groups was estimated through the Concordance index (C-index) (19). Model fitting was done for 1000 bootstraps. The C-index fluctuates from 0.5 to 1, with 1 representing the highest discrimination ability. The time-dependent (tROC) and ROC curves were implemented to verify the prediction accuracy.

A calibration curved line reflecting the relevance between the predicted and the observed survival probability was used to assess the calibration of the model (20). A calibration plot that aligns closely to the 45-degree line indicates an ideal curve, which signifies strong agreement between predicted and actual outcomes. The closer the calibration curve is to the ideal curve, the more unbiased prediction of the model. Finally, a decision curve analysis (DCA) was conducted to assess the net benefit and potential clinical usefulness based on threshold probability. The DCA used the threshold probability to determine the net benefit, which was defined as the difference between the proportion of true positives and false positives, and was weighted by the relative costs of false negatives and false positives (21).

### 2.6 Classification of the risk groups by the model

In the training cohort, the total nomogram score of each patient was calculated. Based on an optimal cut-off determined by X-tile software (22), we stratified patients into two risk groups: high-risk and low-risk. The differences in overall survival (OS) between the high-risk and low-risk groups were assessed using Kaplan-Meier survival analysis and a log-rank test.

### 2.7 Statistical analysis

Kaplan–Meier method was used for survival analysis, and differences between curves were tested by log-rank test. Risk factors of OS was determined by univariate and multivariate cox regression models. The optimal cut-off value of the model scores was calculated through the X-tile software. X-tile is a valuable tool for outcome-based cut-point optimization. The X-tile program for grouping uses each number between the range of the model scores as the cutoff; Then, the χ2 score and P value are calculated using the number as the cutoff; Finally, the number with the maximum χ2 score and the minimum P value will be used as the final cutoff. We deployed SPSS program (version 26.0), R software (version 3.5.3) and X-tile software to analyze data. We judged a *p*-value below 0.05 as statistically significant.

## 3 Results

### 3.1 Comparison of baseline characteristics

A total of 429 IMPC cases were retrieved from the SEER database, while 102 IMPC patients were recruited from the Xijing Hospital. The demographic and clinicopathological information are summarized in **Table 1**. The age, ethnicity, surgery, staging, marital status and ER status differed between cohorts (*p* < 0.05). Compared to the training cohort, N3 and ER-positive patients with a lower proportion characterized the validation cohort. On the other hand, the external cohort was characterized by a higher number of Asian patients, mastectomies, T2 tumors and married patients. The median follow-up was 61 months (IQR, 39-91 months) for the training cohort and 60.5 months (IQR, 53-98 months) for the external cohort.

**Table 1:** Demographic and clinicopathologic features of the training cohort and external validation cohort.

Characteristics	Training cohort (n=429), n (%)	External validation cohort (n=102), n (%)	*P* value
Age	55 (47-63)	49 (42-55)	<0.001
Race			<0.001
White	321 (74.83)	1 (0.98)	
Black	69 (16.08)	2 (1.96)	
Asian and other	39 (9.09)	99 (97.06)	
Laterality			0.110
Left	227 (52.91)	45 (44.12)	
Right	202 (47.09)	57 (55.88)	
Grade			0.173
1	28 (6.53)	12 (11.76)	
2	226 (52.68)	48 (47.06)	
3	175 (40.79)	42 (41.18)	
Examined lymph nodes	6 (2-14)	9 (4-19)	0.091
Positive lymph nodes	1 (0-3)	1 (0-4)	0.544
Surgery			<0.001
No	11 (2.56)	1 (0.98)	
BCS	209 (48.72)	29 (28.43)	
Mastectomy	209 (48.72)	72 (70.59)	
Radiation therapy			0.704
No	194 (45.22)	44 (43.14)	
Yes	235 (54.78)	58 (56.86)	
Chemotherapy			0.731
No	155 (36.13)	35 (34.31)	
Yes	274 (63.87)	67 (65.69)	
Marital status			<0.001
Unmarried	171 (39.86)	20 (19.61)	
Married	258 (60.14)	82 (80.39)	
T stage			0.009
T1	229 (53.38)	40 (39.22)	
T2	141 (32.87)	52 (50.98)	
T3	47 (10.96)	8 (7.84)	
T4	12 (2.79)	2 (1.96)	
N stage			0.027
N0	186 (43.36)	49 (48.04)	
N1	141 (32.87)	42 (41.18)	
N2	58 (13.52)	8 (7.84)	
N3	44 (10.25)	3 (2.94)	
ER			0.044
Negative	55 (12.82)	21 (20.59)	
Positive	374 (87.18)	81, (79.41)	
PR			0.536
Negative	97 (22.61)	26 (25.49)	
Positive	332 (77.39)	76 (74.51)	

### 3.2 Independent prognostic variables in the training cohort

The univariate analysis indicated that marital status, ethnicity, surgery, staging, positive lymph nodes, ER and PR were prognostic factors for OS in non-metastatic IMPC (all *p* < 0.05). Based on these factors screened through univariate analysis, five independent risk factors were identified through multivariate analysis, including race, surgery, positive lymph nodes, T stage, and ER status. The results are summarized in **Table 2**.

**Table 2:** Univariable and multivariable Cox analysis for predicting overall survival in non-metastatic IMPC in training cohort.

Characteristics	Univariate analysis		Multivariate analysis	
	Hazard ratio (95% CI)	P value	Hazard ratio (95% CI)	P value
Age	1.527 (0.896-2.602)	0.120		
Race				
White				
Black	2.220 (1.149-4.289)	**0.018**	1.866 (1.010-3.779)	**0.047**
Asian and other	0.457 (0.109-1.921)	0.285	0.357 (0.079-1.626)	0.183
Laterality				
Left				
Right	0.869 (0.471-1.605)	0.655		
Grade				
1				
2	2.350 (0.011-3.870)	0.594		
3	4.595 (2.478-9.805)	0.572		
Surgery				
No				
BCS	0.137 (0.039-0.480)	**0.002**	0.164 (0.037-0.722)	**0.017**
Mastectomy	0.247 (0.074-0.827)	**0.023**	0.124 (0.031-0.498)	**0.003**
Radiation therapy				
No				
Yes	0.824 (0.453-1.500)	0.527		
Chemotherapy				
No				
Yes	1.240 (0.655-2.349)	0.509		
Examined lymph nodes	1.021 (0.991-1.053)	0.172		
Positive lymph nodes	1.207 (1.105-1.319)	**<0.001**	1.128 (1.009-1.282)	**0.027**
Marital status				
Unmarried				
Married	0.497 (0.271-0.910)	**0.023**	0.743 (0.385-1.432)	0.375
T stage				
T1				
T2	1.370 (0.627-2.990)	0.430	0.949 (0.399-2.256)	0.906
T3	4.859 (2.210-10.683)	**<0.001**	2.149 (0.754-6.124)	0.152
T4	12.314 (4.739-31.998)	**<0.001**	3.988 (1.032-15.410)	**0.045**
N stage				
N0				
N1	2.025 (0.864-4.747)	0.104	1.935 (0.769-4.868)	0.161
N2	2.487 (0.883-7.004)	0.085	1.951 (0.554-6.864)	0.298
N3	7.412 (3.242-16.947)	**<0.001**	2.636 (0.507-13.707)	0.249
ER				
Negative				
Positive	0.316 (0.167-0.599)	**<0.001**	0.241 (0.073-0.790)	**0.019**
PR				
Negative				
Positive	0.481 (0.261-0.888)	**0.019**	1.421 (0.466-4.337)	0.537

The bold values mean P< 0.05.

### 3.3 Establishment of the nomogram of prognosis

**Figure 1:**
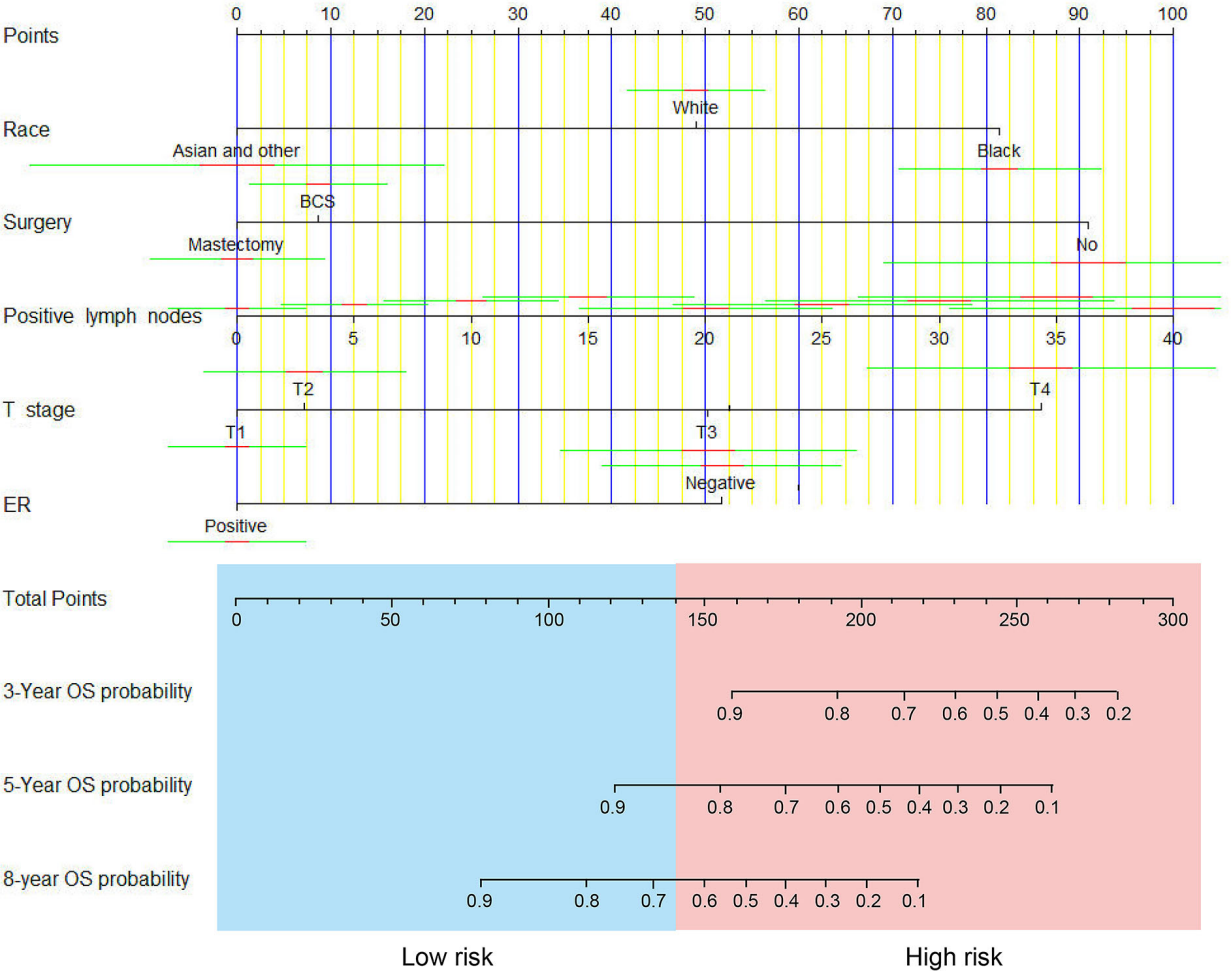
A nomogram for predicting 3-, 5- and 8-year overall survival (OS) of patients with non-metastatic IMPC.

The nomogram predicting survival time based on the 5 independent risk factors, including race (White, Black, Asian or other), surgery (none, breast conservative surgery (BCS) or mastectomy), number of positive lymph nodes, T stage (T1, T2, T3 or T4) and ER status (negative or positive) is illustrated in **Figure 1**. The risk score for each independent prognostic indicator was calculated by sketching a vertical line from the independent variable. The total risk was then calculated by considering the scores obtained for each variable. The OS at 3, 5 and 8 years was calculated by sketching a line from the total points axis to the corresponding survival axis.

### 3.4 Verification of the model’s performance

**Figure 2:**
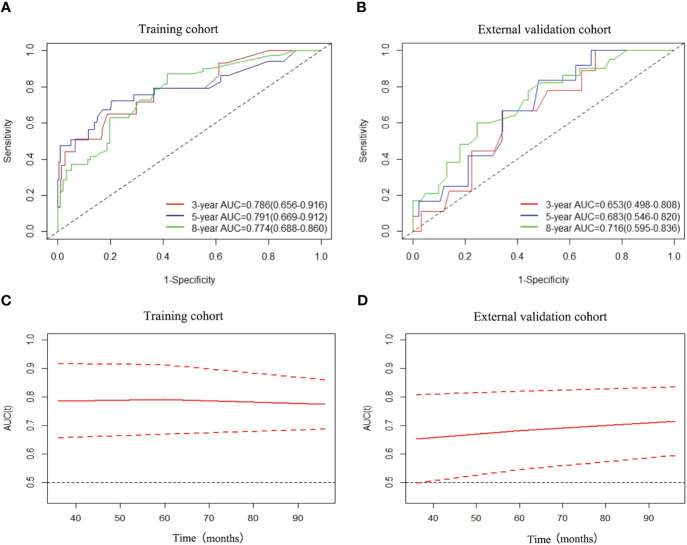
ROC curves and AUCs of nomogram for predicting 3-year, 5-year and 8-year OS in the training cohort **(A)** and external validation cohort **(B)**, and time-dependent AUC values of nomogram in the training cohort **(C)** and external validation cohort **(D)**.

Regarding OS, the C-index showed a good prognostic discrimination for both training (0.766, 95% CI, 0.682-0.850) and validation cohorts (0.694, 95% CI, 0.527-0.861). The AUC values were 0.786 (95%CI: 0.656-0.916) at 3 years, 0.791 (95%CI: 0.669-0.912) at 5 years and 0.774 (95%CI: 0.688-0.860) at 8 years for the training cohort. The AUC values were 0.653 (95%CI: 0.498-0.808) at 3 years, 0.683 (95%CI: 0.546-0.820) at 5 years and 0.716 (95%CI: 0.595-0.836) at 8 years for the external cohort (**Figures 2A, B**). The time-dependent AUC values for the training cohort fluctuated around 0.8, while for the external cohort, they fluctuated around 0.7. This demonstrates excellent performance and discrimination of the model (**Figures 2C, D**).

**Figure 3:**
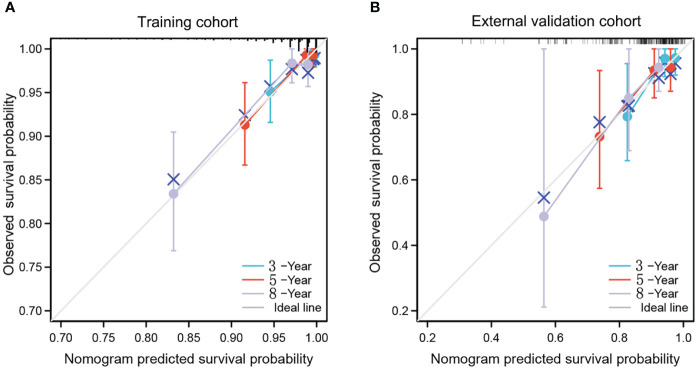
calibration curves of nomogram for predicting 3-year, 5-year and 8-year OS in the training cohort **(A)** and external validation cohort **(B)**. The x-axis indicates the predicted survival probability, and the y-axis indicates the actual survival probability. The 45-degree line (gray line) indicates that the prediction agrees with actuality.

**Figure 4:**
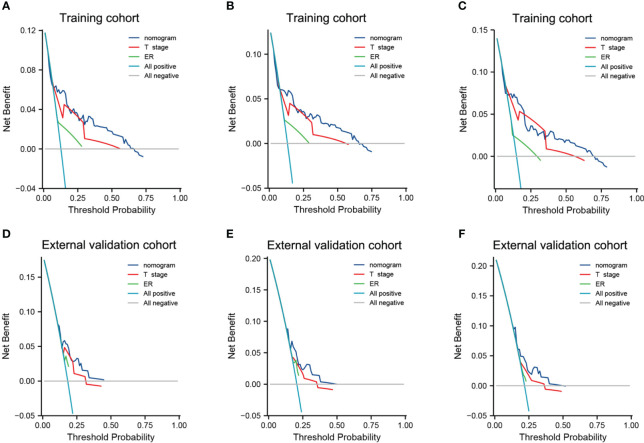
Decision curve analysis of the nomogram and single independent predictors for predicting the 3-year OS **(A, D)**, 5-year OS **(B, E)** and 8-year OS **(C, F)** in the training cohort and external validation cohort, respectively. Light green line: net benefit of a strategy of treating all IMPC patients. Gray line: net benefit of treating no IMPC patients. Colored lines: net benefit of a strategy of treating patients according to the nomogram, T stage and ER.

The calibration curves indicated a satisfactory agreement between predictions and observed OS in both cohorts (**Figures 3A, B**). The 3-, 5- and 8-year decision curve analysis showed that the net benefit of the nomogram was superior to the net benefit of single factors almost across the entire range of threshold probabilities, indicating the nomogram had a favorable performance in predicting survival (**Figure 4**).

### 3.5 Risk stratification

**Figure 5:**
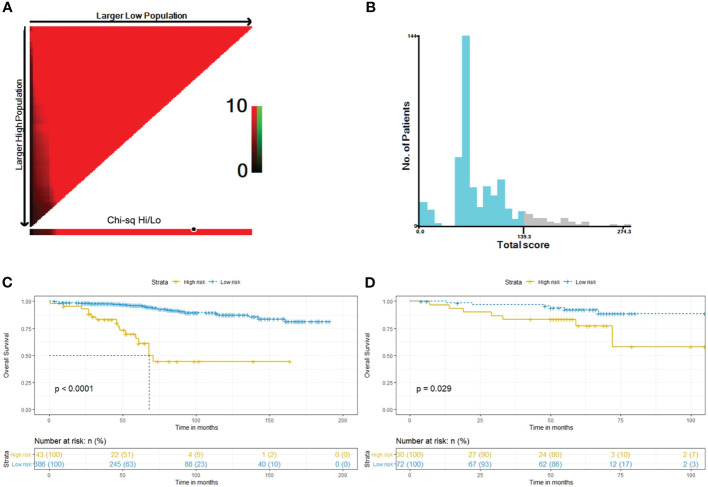
Determination of best cut-off value of total sum score by the X-tile software **(A, B)**. Kaplan–Meier curves of OS for risk stratification in the training cohort **(C)** and the external validation cohort **(D)**.

Considering the total sum score, the optimal cut-off value was 139.3. Patients with a total sum score below this cut-off were identified as low risk patients, whereas those with total sum scores above or equal to the cut-off were identified as high risk patients (**Figures 5A, B**). In the training cohort, we classified 43 high-risk patients and 386 low-risk patients. In the validation cohort, we classified 27 high-risk patients and 75 low-risk patients. The Kaplan-Meier survival curves revealed a different survival between groups in both cohorts (*p* < 0.001 and *p* = 0.0074) (**Figures 5C, D**).

## 4 Discussion

IMPC is a heterogeneous form of breast cancer. Respect to IDC, IMPC is associated with a worse prognosis (23–25). IMPC has different characteristics from other common breast cancer histological subtypes. As a result, several other factors may affect the prognosis of IMPC besides the tumor stage. We developed a novel risk stratification nomogram to predict the OS in non-metastatic IMPC. Clinical information of IMPC individuals were retrieved from the SEER database, which covers about 30% of all cancers in the US. However, since the US and Chinese populations differ genetically and demographically, we also validated the model on a cohort of IMPC cases obtained from the Xijing Hospital, one of the biggest hospitals in northwest China, to confirm the applicability and accuracy of the stratification nomogram.

After performing univariate and multivariate analyses, we identified 5 independent risk factors for OS, including race, surgery, positive lymph nodes, T stage and ER status. These factors were used to develop a predictive nomogram. Consistent with previous studies, we identified a strong association between positive lymph nodes and worse survival outcomes. Therefore, positive lymph nodes were given the highest weighting and incorporated as a continuous variable to improve the prediction accuracy of the nomogram. Micropapillary carcinomas of the breast have a well-recognized lymphovascular tropism that leads to more patients presenting with clinically disease-positive lymph nodes (26). The breast surgeon must be aware of the lymphotropic behavior of this subtype and the high prevalence of lymph node involvement in such patients, and therefore focus on rigorous axillary assessment. One must not forget that, despite having a more aggressive biological profile, IMPC has demonstrated no difference in survival when compared to other histological subtypes, and treatment should conform to international guidelines with an emphasis on nodal staging (27).

Compared with IDC, IMPC has a higher incidence of lymph involvement ranging from 60% to 90% (4, 28–30). We described that the incidence of lymph node involvement was higher in the training cohort (56.64%) than in the validation cohort (51.96%), possibly due to variations in the T-stage, ER status, age, and race between the 2 groups. Meanwhile, our analysis indicated that Asian patients with non-metastatic IMPC had a better prognosis than black patients, possibly due to the lower rate of lymphatic metastasis. Though there are no reports of such findings in non-metastatic IMPC, some studies on IDC also showed that Asian breast cancer patients tend to have a better prognosis (31, 32).

The individuals pertaining to the training cohort reported a lower T stage compared to those in the external cohort. Due to a small tumor size, more patients in the training cohort received BCS respect to the other cohort (48.72% versus 28.43%, *p*<0.001). Interestingly, although previous studies showed that patients treated with BCS and mastectomy had the same prognosis (33, 34), in our study, breast mastectomy was an independent high-risk indicator for OS more than BCS, possibly since patients who underwent BCS tended to have a lower T-stage. Furthermore, various studies also demonstrated that ER expression is an important predictor for OS, recurrence-free survival and breast cancer-specific survival in IMPC patients (35, 36). Our study also identified ER status as an important prognostic indicator.

Inconsistent with previously published work, age was not an independent risk factor for OS. Younger patients often tend to have more aggressive advanced breast cancer than older patients (37, 38). However, the proportion of ER-positive patients with breast cancer was relatively high in our cohort. Since ER status greatly impacts OS, age was not identified as an independent risk factor.

Individuals from the training were significantly older than those in the validation cohort (55 years versus 49 years, p < 0.001). In the SEER database, the median age of women with breast cancer is 61 years (39). However, in China, the age of women with breast carcinoma is frequently reported from 45 to 55 years. The younger age in Chinese patients could be due to the birth cohort effect, variations in menstrual and reproductive patterns or other environmental factors (40). Since Chinese patients were younger than those in US, they were likely to present with an advanced breast cancer. Notably, our analysis showed no difference regarding 5-year OS between cohorts. In low-risk groups, the 5-year OS was higher in the training cohort. In high-risk groups, the 5-year OS was lower in the training cohort.

The AUC, C-index and calibration results showed that the proposed nomogram achieved good discrimination and accuracy in both cohorts. The DCA confirmed that our nomogram could significantly benefit decision-making compared to single-factor models. Additionally, the nomogram could accurately stratify the IMPC patients in high-risk and low-risk groups. According to this stratification, clinicians can more accurately predict the survival time and therefore optimize the follow-up and treatment according to the patient’s needs.

Our study has some limitations. The retrospective design and the small sample size in our study could have introduced selection bias, limiting the generalizability of the research findings. Moreover, the SEER database lacked detailed information about important predictive factors for OS, such as chemotherapy treatment, lymphovascular invasion, genetic mutations, and the proportion of the IMPC subtype within the breast sample. Larger prospective studies are recommended to identify the impact of other variables on OS and validate the nomogram’s predictive accuracy.

## 5 Conclusions

We developed a nomogram predicting OS specifically for the IMPC breast cancer subtype. Our novel prognostic nomogram for non-metastatic IMPC patients achieved satisfactory discrimination and predictive accuracy in both cohorts. Clinicians could use the nomogram to optimize the follow-up and treatment in accordance with patient’s specific risk factors.

## Data availability statement

The datasets presented in this study can be found in online repositories. The names of the repository/repositories and accession number(s) can be found in the article/**Supplementary Material**.

## Author contributions

MZ and YQ completed the manuscript, and NH prepared **Tables 1** and **2**. FJ and ZZ prepared **Figures 1** to **5**. JZ revised the paper. All authors contributed to the article and approved the submitted version.
